# Reduction of Neuroinflammation by δ-Opioids Via STAT3-Dependent Pathway in Chronic Glaucoma Model

**DOI:** 10.3389/fphar.2021.601404

**Published:** 2021-02-08

**Authors:** Shahid Husain, Syed A. H. Zaidi, Sudha Singh, Wendy Guzman, Shikhar Mehrotra

**Affiliations:** ^1^ Department of Ophthalmology, Storm Eye Institute, Medical University of South Carolina, Charleston, SC, United States; ^2^ Department of Surgery, Hollings Cancer Center, Medical University of South Carolina, Charleston, SC, United States

**Keywords:** protein acetylation, transcription factors, opioids, glaucoma, neuroinflammantion

## Abstract

The main objective of this study was to determine the inhibition of pro-inflammatory cytokines and their associated signaling molecules by δ-opioid receptor activation by a selective ligand, SNC-121 in chronic rat glaucoma model. Intraocular pressure was raised in rat eyes by injecting 2 M hypertonic saline into the limbal veins. SNC-121 (1 mg/kg; i. p) or Stattic (5 mg/kg; i. p) was administered in Brown Norway rats daily for 7 days. The mRNA expression of IL-1β, TNF-α, Fas, IL-6, leukemia inhibitory factor, and IFN-γ was increased significantly in the retina of ocular hypertensive animals at day 7, post injury. Administration of SNC-121 (1 mg/kg; i. p. injection) for 7 days (once a day) completely inhibited the increase in the mRNA and protein expression of pro-inflammatory cytokines. Mechanistically, we provide data showing a significant increase in the phosphorylation of STAT3 at tyrosine 705 whereas a moderate but significant increase in the total STAT3 protein expression was also seen in the retina of ocular hypertensive animals. Data illustrated that SNC-121 administration completely abrogated ocular hypertension-induced increase in STAT3^Y705^ phosphorylation. Interestingly, acetylation of STAT3 at lysine 685 (AcK685) was reduced in ocular hypertensive animals and subsequently increased significantly by SNC-121 treatment. Stattic, a selective STAT3 inhibitor, administration resulted in a complete attenuation in the production of IL-1β and IL-6 in ocular hypertensive animals. In conclusion, δ-opioid receptor activation suppressed the phosphorylation of STAT3 at tyrosine 705 and increased acetylation at lysine 686 and these posttranslational modifications can regulate the production of some but not all pro-inflammatory cytokines in response to glaucomatous injury.

## Introduction

Glaucoma is the second leading cause of blindness worldwide in which retinal ganglion cells (RGCs) die slowly and progressively over a prolonged period of time. Glaucoma is now considered to be a multi-factorial disease in which biomechanical stress (Boland and Quigley, 2007), epigenetic changes (Crosson et al., 2010; Pelzel et al., 2010; Alsarraf et al., 2014b; Zaidi et al., 2020), mitochondrial dysfunction (Osborne et al., 2016), deprivation of neurotrophic factors (Chitranshi et al., 2018), oxidative stress (Benoist d’Azy et al., 2016), and neuroinflammation (Husain et al., 2012a; Husain et al., 2012b; Abdul et al., 2013; Williams et al., 2017; Adornetto et al., 2019), play critical roles. Studies have shown elevated levels of pro-inflammatory cytokines in human glaucoma patients (Zenkel et al., 2010; Chua et al., 2012; Ohira et al., 2016) and experimental glaucoma models (Husain et al., 2012a; Abdul et al., 2013; Wilson et al., 2015). Additionally, ocular inflammation which has been a prominent feature of uveitis can also lead to inflammatory or uveitic glaucoma (Bodh et al., 2011; Siddique et al., 2013; Baneke et al., 2016; Ohira et al., 2016). A limited number of glaucoma patients have also shown optic disk hemorrhage (Airaksinen and Heijl, 1983; Drance, 1989; Gordon and Piltz-Seymour, 1997), which indicates a breach of the blood-retina barrier. As a result, circulating immune cells can enter the retina and nerve fiber layer where the hemorrhage has been observed most prominently (Drance, 1989; Choi et al., 2007; Hwang et al., 2014). These observations advocate for a potential role of neuroinflammation in glaucoma, however, it is not clear if neuroinflammation is a primary cause or a compensatory response or it is entirely a secondary event during glaucoma pathology that can slowly and progressively damage RGCs.

Epigenetic modifications appear to be a promising novel approach to modulate cellular function in neurodegenerative diseases (Didonna and Opal, 2015; Neal and Richardson, 2018), however their roles in the regulation of neuroinflammation in glaucoma remains unknown. In general, epigenetic changes control gene expression via histone acetylation, DNA methylation, and non-coding RNAs. Protein acetylation is a dynamic process, which is controlled by two classes of enzymes called histone acetyltransferase (HATs) and histone deacetylates (HDACs). Studies have shown impairment in the acetylation homeostasis in numerous neurodegenerative diseases including glaucoma (Ferrante et al., 2003; Rouaux et al., 2003; Ryu et al., 2005; Saha and Pahan, 2006; Haberland et al., 2009; Crosson et al., 2010; Pelzel et al., 2010; Alsarraf et al., 2014a; Alsarraf et al., 2014b; Schmitt et al., 2014; Lebrun-Julien and Suter, 2015; Alkozi et al., 2017; Berson et al., 2018). Earlier, we have shown that δ-opioid receptor activation by a selective ligand (i.e., SNC-121) provides RGC neuroprotection (Abdul et al., 2013; Husain et al., 2014; Husain et al., 2017; Husain, 2018). We have also shown that activation of δ-opioid receptor reduces oxidative stress and proinflammatory cytokines in acute ischemia/reperfusion, chronic rat glaucoma model, and in the optic nerve head (ONH) astrocytes (Husain et al., 2009; Abdul et al., 2013; Akhter et al., 2013b; Husain et al., 2014). Studies have also shown that Sigma-1 receptor agonist reduced intraocular pressure in normotensive rabbit(Bucolo et al., 1999). Sigma-1 receptor is a ligand-operated chaperone that modulates neurotransmission by interacting with multiple protein partners, including the *µ*-opioid receptor and sigma-1 antagonists are able to induce opioid analgesia by enhancing the action of endogenous opioid peptides of immune origin during inflammation (Tejada et al., 2018). Additionally, Sigma-1 receptor modulate neuroinflammation in osteoarthritis (Carcole et al., 2019). However, the interaction of sigma-1 and δ-opioid receptors was not determined in the current study. More recently, we have shown that SNC-121 increased histone H2B, H3, and H4 acetylation and reduced the activities and expression of class I and IIb histone deacetylates (HDACs) in ONH astrocytes (Zaidi.et al., 2020). However, it remains in question if protein acetylation of certain transcription factors plays any crucial role in the regulation of pro-inflammatory cytokines in glaucoma.

Based on our data we hypothesize that δ-opioids induced acetylation of transcription factor, such as STAT3, can regulate the production of pro-inflammatory cytokines under glaucomatous conditions. To the best of our knowledge this is the first report in which we have shown that δ-opioid receptor activation regulates phosphorylation of STAT3 at tyrosine 705 which subsequently plays key roles in the regulation of neuroinflammation during glaucoma progression.

## Materials and Methods

### Animals

Equal numbers of adult male and female Brown Norway rats (2–5 months of age; 150–350 g) were used in this study. These animals were obtained from Charles River laboratory (San Diego, CA). Rats were maintained under a cycle of 12-hours light and 12-h dark throughout the studies. All animal handling was performed in accordance with the ARVO Statement for the Use of Animals in Ophthalmic and Vision Research. The study protocol was approved by the Animal Care and Use Committee at the Medical University of South Carolina. Delta (δ)-opioid-receptor agonist, SNC-121 (1 mg/kg body weight; Santa Cruz Biotechnology, Dallas, TX), was dissolved in PBS. Stattic (5 mg/kg body weight) (Tocris, Minneapolis, MN) was dissolved in 75% DMSO. Drugs or vehicle were administered individually in Brown Norway rats intraperitoneally (i.p.) 30 min post hypertonic saline injection. The drug administration (150–200 µL) was continued for 7 days, once daily at the same time (i.e., 9 am–11 am).

### Development of Glaucoma Model by Hypertonic Saline Injection

Intraocular pressure (IOP) was raised in Brown Norway rats by injecting 50 µL of 2 M hypertonic saline into the limbal venous system as described earlier (Abdul et al., 2013; Husain et al., 2014; Husain et al., 2017). IOP was recorded at day 0 (prior to injury), day 3 and 7 (post injury). Animals that had elevation in IOP more than 25% were included in the data analysis.

### RNA Extraction and Real Time-Polymerase Chain Reaction

RNA from the retina was isolated using TRIzol reagent (Life Technologies, Grand Island, NY) as described previously (Zaidi.et al., 2020). Purity and concentration of the isolated RNA was determined using Nanodrop. Total RNA (1 μg) was used to prepare cDNA using iScript cDNA synthesis Kit (Bio-Rad Laboratories, Inc., Hercules, CA, United States) according to the manufacturer's manual. The changes in mRNA levels for IL-1β, TNF-α, Fas, IL-6, LIF, IFN-γ, STAT1, STAT2, and STAT3 were analyzed by quantitative Real Time (RT) PCR (Bio-Rad iCycler system, Hercules, CA) using specific primers synthesized from Integrated DNA technologies (Coralville, IA). The sequences of specific primer used for each gene are listed in Table 1. Conditions for qRT- PCR were: 95°C for 30 s followed by two-step 40 cycles of 95°C for 10 s and 60°C for 30 s. The relative gene expression is calculated based on the comparative threshold cycle (ΔΔCt) method. Expression levels for all genes were normalized to the mean value of housekeeping gene *ß*-actin. We have used 4–9 animals in each group of treatment and a total of 19 animals were used for qRT-PCR.

**TABLE 1:** Primer sequences used for quantitative real time-polymerase chain reaction in the current study.

Primer	Direction	Sequence
IL-1β	Forward	TGA​GGC​TGA​CAG​ACC​CCA​AAA​GAT
IL-1β	Reverse	GCT​CCA​CGG​GCA​AGA​CAT​AGG​TAG
IL-6	Forward	TGT​ATG​AAC​AGC​GAT​GAT​G
IL-6	Reverse	AGAAGACCAGAGCAGATT
TNF-α	Forward	AAA​TGG​GCT​CCC​TCT​CAT​CAG​TTC
TNF-α	Reverse	TCT​GCT​TGG​TGG​TTT​GCT​ACG​AC
IFN-γ	Forward	CACGCCGCGTCTTGGT
IFN-γ	Reverse	TCT​AGG​CTT​TCA​ATG​AGT​GTG​CC
LIF	Forward	ACC​AGA​TCA​AGA​GTC​AAC​TG
LIF	Reverse	CCT​TGA​GCT​GTG​TAA​TAG​GA
FAS	Forward	CTT​GGG​TGC​CGA​TTA​CAA​CC
FAS	Reverse	GCC​CTC​CCG​TAC​ACT​CAC​TC
STAT1	Forward	AGA​GCG​ACC​AGA​AAC​AGG​AA
STAT1	Reverse	GCT​CTC​TGC​AAC​AAT​GGT​GA
STAT2	Forward	TGG​TTC​AAC​ATG​CTC​AGC​TC
STAT2	Reverse	GCT​CTC​TCG​CTT​GCT​GAA​GT
STAT3	Forward	AGT​TCT​CGT​CCA​CCA​CCA​AG
STAT3	Reverse	CTA​GCC​AGA​CCC​AGA​ACG​AG
β-actin	Forward	ATG​GTC​AAC​CCC​ACC​GTG​T
β-actin	Reverse	CGT​GTG​AAG​TCA​CCA​CCC​T

### Immunohistochemistry

Eyes were enucleated on day 7, post hypertonic saline injection and placed in 4% PFA for 12 h at 4°C. The retinas were then transferred to 30% sucrose for 12 h followed by embedding the retinas in OCT on dry ice. Cryosections were cut at −30°C and stored at −20°C until used for immunohistochemistry. Retina sections were rinsed with Tris-buffered saline (TBS) at room temperature followed by permeabilization using 0.5% Triton-X-100 in TBS. The tissues were blocked with 5% BSA for 1 h followed by incubation with primary antibodies (anti-IL-1β, anti-IL-6, or anti-phospho-STAT3) for 16 h at 4°C. After washing, the tissues were incubated with secondary antibody (Alexa Fluor 488 or Rhodamine) for 1 h at room temperature. The details of antibodies dilution and commercial sources are provided in Table 2. The sections were observed under a bright-field microscope equipped with epifluorescence, and digitized images were captured by a digital camera (Zeiss) as described earlier (Husain et al., 2012a; Abdul et al., 2013; Husain et al., 2014; Husain et al., 2017). We have used four animals in each group of treatment and a total of 12 animals were used for Immunohistochemistry.

**TABLE 2:** List of antibodies, vendors, catalog number, and dilutions used in the current study.

Antibody	Company	Catalog No	Application	
			IHC	WB
Anti-acetyl STAT3 lys 685	Cell signaling technology	2,523	1:1,000
Anti-β actin	Sigma aldrich	A5316	1:2000
Anti-IL-6 (10E5)	Invitrogen	ARC0962	1:100
Anti-IL1β (H-153)	Santa cruz biotechnology	Sc-7884	1:20
Anti-phospho STAT3 Tyr 705 (D3A7)	Cell signaling technology	9,154	1:100	1:1,000
Anti- STAT3 (124H6)	Cell signaling technology	9,139	1:2000
Anti-rabbit IgG, HRP-linked	Cell signaling technology	7,074	1:5,000
Anti-mouse IgG, HRP-linked	Cell signaling technology	7,076	1:5,000
Alexa fluor 488 anti mouse	Jackson immunoresearch	111–545–044	1:500
Alexa fluor 488 anti rabbit	Jackson immunoresearch	111–545–144	1:500
Rhodamine red anti rabbit	Jackson immunoresearch	711–295–152	1:500
Rhodamine red anti mouse	Jackson immunoresearch	715–025–151	1:500

### Protein Extraction and Western Blotting

Protein extracts were prepared by homogenizing the retina using handheld micro-grinder in lysis buffer (50 mM Tris-HCl buffer, pH 8.0 containing 10 mM EDTA, 0.5% sodium deoxycholate, 0.5 mM sodium orthovanadate, 1%Triton X-100, and Halt’s protease inhibitor cocktail). Protein lysate were then centrifuged at 10,000 rpm for 10 min at 4°C and total proteins in the supernatant were quantified by Bradford Protein Assay (BioRad, Hercules, CA). Total protein (10–20 μg) for each sample were separated by NuPAGE 10% Bis-Tris Gels (Thermo Fisher Scientific, Carlsbad, CA) and proteins were transferred to a nitrocellulose membrane. The membranes were blocked with 5% BSA or non-fat dry milk in a Tris-buffer saline containing 1% Tween-20 for 1 h. Membranes were incubated with primary antibodies (anti-phospho STAT3 Tyr 705, anti-acetyl-STAT3 Lys K685, or anti-STAT3) for 16 h at 4°C. Membranes were probed with horseradish peroxidase-(HRP) linked secondary antibody, and visualized by enhanced chemiluminescence reagent (ECL, Super Signal, Thermo Scientific, Rockford, IL) on Biorad Versadoc imaging system (Biorad, Hercules, CA). All membranes were reprobed with *ß*-actin to ensure equal protein loading. Band intensities of each protein of interest were measured by densitometry as arbitrary units and normalized with *ß*-actin as an internal loading control. The details for the antibody dilution and sources are provided in Table 2. We have used 6–9 animals in each group of treatment and a total of 21 animals were used in protein estimations by Western blotting.

### Statistical Analysis

Data are presented in this manuscript as ± standard error of mean (SEM) from three or more independent experiments as indicated by n. Where “n” refers to biological replicates. Statistical analysis was performed using one-way ANOVA followed by a post hoc Tukey test for multiple comparisons (GraphPad Software, Inc., San Diego, CA). A *p* value ≤0.05 was considered significant.

## Results

### Changes in the Intraocular Pressure in Response to Glaucomatous Injury

Intraocular pressure (IOP) was measured at day 0, 3, and 7, pre- and post-glaucomatous injury. As shown in Table 3, a significant increase in the IOP was seen as early as day 3, which was further increased significantly at day 7, post injury. Similar to our previous studies SNC-121 had no effects on IOP (Abdul et al., 2013; Husain et al., 2014). Additionally, we have not seen any effects of Stattic treatment on IOP.

**TABLE 3:** Measurements of IOP in normal, ocular hypertensive, SNC-121, and stattic treated ocular hypertensive animals.

Days	0-Day	3-Days	7-Days
Normal animals	17.3 ± 0.58	17.9 ± 0.40	19.3 ± 0.47
Ocular hypertensive animals	16.8 ± 0.52	22.3 ± 0.92**	25.8 ± 0.59****
Ocular hypertensive animals + SNC 121 (1 mg/kg)	16.9 ± 0.60	20.8 ± 1.0*	25.5 ± 0.6****
Ocular hypertensive animals + Stattic (5 mg/kg)	17.3 ± 0.3	19.2 ± 0.3	22.0 ± 0.6*

Data are expressed as mean ± SE; *n* = 8–12. Asterisk (*) indicates significant difference from normal animals at each time point. *p = 0.02, **p = 0.0025, and ****p = 0.001.

### Regulation of Pro-inflammatory Cytokines by δ-Opioid Receptor Activation in Rat Glaucoma Model

**FIGURE 1:**
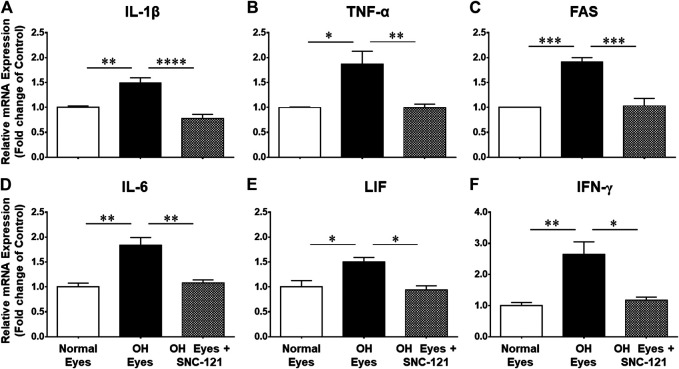
Changes in mRNA expression of pro-inflammatory cytokines in response to ocular hypertension and SNC-121 treatment. Intraocular pressure (IOP) was raised by 2.0 M hypertonic saline injection followed by δ-opioid receptor agonist (1 mg/kg; i. p injections) treatment for 7 days, once a day. The retinas were collected at day 7, post hypertonic saline injection and mRNA was analyzed for IL-1β **(A)**, TNF-α **(B)**, FAS **(C)**, IL-6 **(D)**, LIF **(E)**, and IFN-γ **(F)** using cDNA that was synthesized from 1 µg total RNA. The relative changes in mRNA levels were measured by quantitative RT-PCR (qRT-PCR) using primers specific for each gene as indicated in Table 1. The qRT-PCR data was normalized using *ß*-actin gene expression as an internal control. The relative expression was calculated based on the comparative threshold cycle (2^−ΔΔCt^) method. Bar graph represents mean data ±SEM. OH Eyes; ocular hypertensive eyes; OH Eyes + SNC-121, ocular hypertensive eyes with SNC-121 treatment. *p < 0.05; **p < 0.01; ***p < 0.001; ****p < 0.0001; *n* = 4–9. In this experiment “n” represent a biological replicate.

**FIGURE 2:**
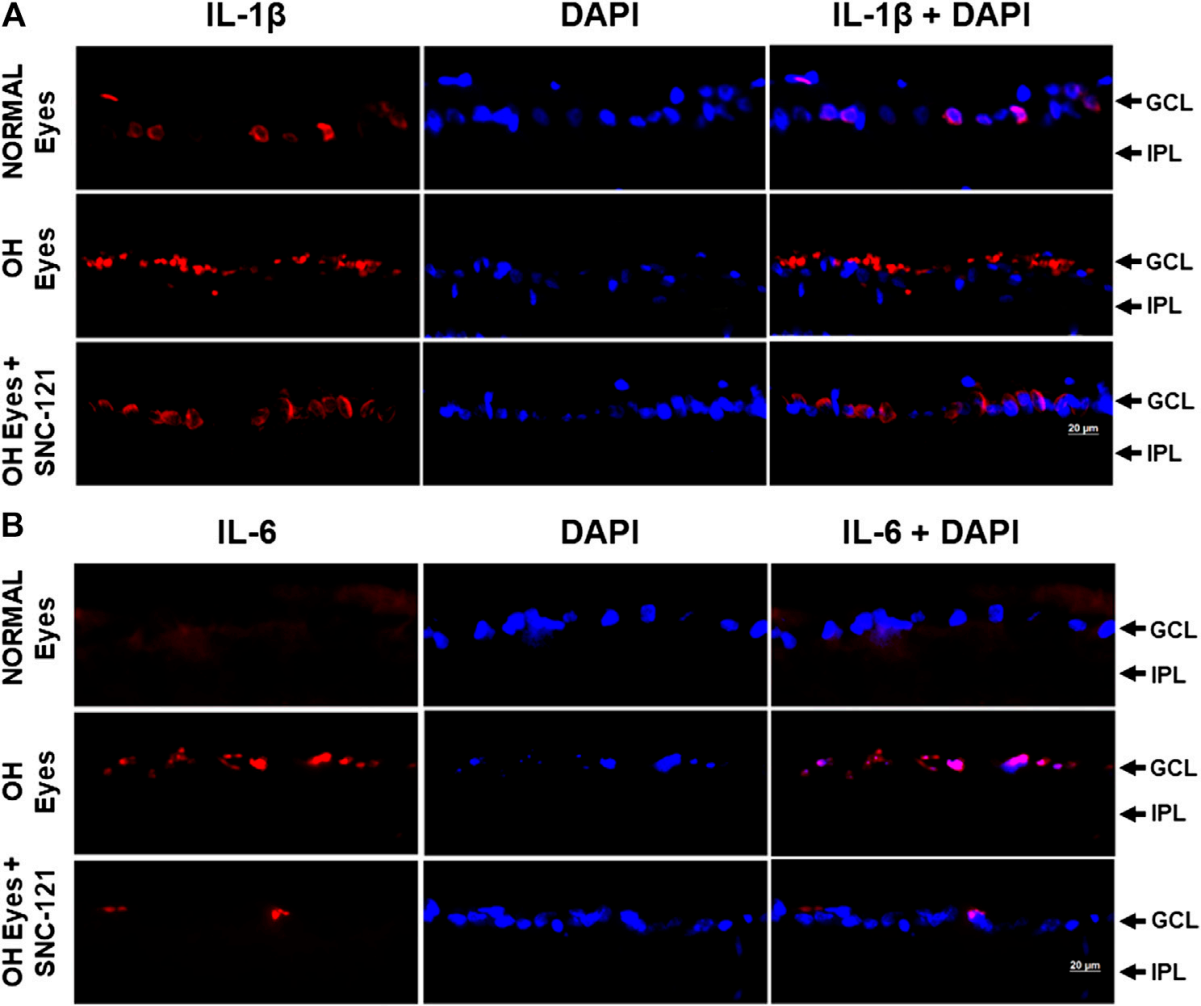
Effects of δ-opioid receptor agonist (SNC-121) treatment on interleukin-1β (IL-1β) **(A)** and interleukin-6 (IL-6) **(B)** production in ocular hypertensive animal. Animals were euthanized and eyes were enucleated at day 7, post hypertonic saline injections. Retina cryosections were immunostained using a selective anti-IL-1β or anti-IL-6 antibody. The nuclei were counterstained with DAPI. Red color indicates staining for IL-1β and IL-6 and blue for the nuclei. There was no positive immunostaining when primary antibodies were omitted (not shown). Data shown here is a representation of at least four independent experiments. We have used four animals in each treatment group. OH Eyes; ocular hypertensive eyes; OH Eyes + SNC-121, ocular hypertensive eyes with SNC-121 treatment, bar size 20 µm.

We determined the regulatory effects of a selective δ-opioid receptor agonist (SNC-121) on the production of pro-inflammatory cytokines and their associated signaling molecules in the retina of ocular hypertensive animals. We chose to measure changes in the mRNA expression of interleukin-1β (IL-1β), tumor necrosis factor-α (TNF-α), Fas, interleukin-6 (IL-6), leukemia inhibitory factor (LIF), and interferon γ (IFN-γ) based on their potential involvement in neurodegenerative diseases. The mRNA expression of IL-1β was increased by 1.49 ± 0.11 fold (*n* = 6–9; *p* < 0.01; Figure 1A), TNF-α by 1.87 ± 0.26 fold (*n* = 4–7; *p* < 0.05; Figure 1B), Fas by 1.92 ± 0.08 fold (*n* = 4; *p* < 0.001; Figure 1C), IL-6 by 1.84 ± 0.15 fold (*n* = 4–6; *p* < 0.01; Figure 1D), LIF by 1.5 ± 0.1 fold (*n* = 4; *p* < 0.05; Figure 1E), and IFN-γ by 2.64 ± 0.41 fold (*n* = 4–7; *p* < 0.01; Figure 1F), at day 7, post injury. Treatment of SNC-121 (1 mg/kg; i. p. injection) for 7 days (once a day) completely inhibited the increase in the mRNA expression of pro-inflammatory cytokines (Figures 1A–D). Earlier, we have shown that elevated TNF-α protein expression was completely blocked by SNC-121 treatment in chronic glaucoma model (Abdul et al., 2013). In the current study, we have also shown that protein expression of IL-1β and IL-6 was remarkably increased in RGC and nerve fiber layers in ocular hypertensive animals (Figure 2), which was completely inhibited in SNC-121 treated ocular hypertensive animals.

### Effects of δ-Opioid Receptor Agonist (SNC-121) on Transcription Factors STATS in Glaucoma Model

**FIGURE 3:**
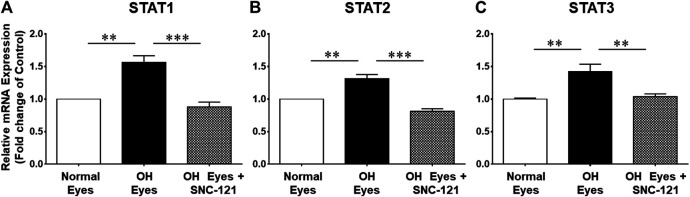
Effects of SNC-121 treatment on mRNA expression of STAT1 **(A)**, STAT2 **(B)**, and STAT3 **(C)**. The retina from normal, ocular hypertensive, and SNC-121 treated ocular hypertensive animals were collected at day 7, post hypertonic saline injection. Complementary DNA was synthesized from 1 µg total RNA extracted from retina. The relative changes in mRNA levels were measured by quantitative RT-PCR (qRT-PCR) using primers specific for each gene as indicated in Table 1. The qRT-PCR data was normalized using *ß*-actin gene expression as an internal control. The relative expression was calculated based on the comparative threshold cycle (2^−ΔΔCt^) method. Bar graph represents mean data ±SEM. OH Eyes; ocular hypertensive eyes; OH Eyes + SNC-121, ocular hypertensive eyes with SNC-121 treatment. **p < 0.01; ***p < 0.001; *n* = 4–9. In this experiment “n” represent a biological replicate.

To learn more about the signaling mechanisms that may be involved in the production of pro-inflammatory cytokines in response to glaucomatous injury, we measured the changes in the mRNA transcript of transcription factors, signal transducer and activator of transcription (e.g., STAT1, STAT2, and STAT3) in the retina of normal and ocular hypertensive animals. As shown in Figure 3, the mRNA expression of STAT1, STAT2, and STAT3 was increased significantly in the retina of ocular hypertensive animals. The mRNA transcripts of STAT1, STAT2, and STAT3 were increased by 1.56 ± 0.11 fold (*n* = 4; *p* < 0.01; Figure 3A), 1.31 ± 0.07 fold (*n* = 4; *p* < 0.01; Figure 3B), and 1.43 ± 0.11 fold (*n* = 7–9; *p* < 0.01; Figure 3C), respectively. Interestingly, up-regulation of STAT1, STAT2, and STAT3 mRNA was blocked in SNC-121 treated ocular hypertensive animals (Figures 3A–C).

### Effects of δ-Opioid Receptor Agonist (SNC-121) on STAT3^Y705^ Phosphorylation

**FIGURE 4:**
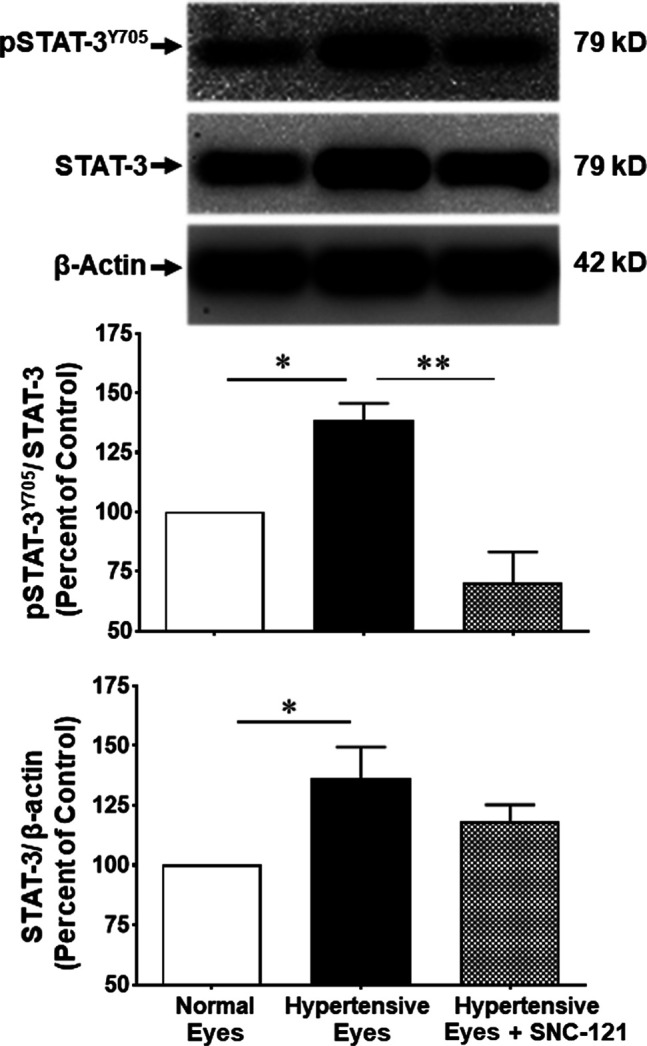
Changes in the phosphorylation of STAT3 (Tyr 705) and total STAT3 by SNC-121 treatment in ocular hypertensive animals. The retina from normal, ocular hypertensive, and SNC-121 treated ocular hypertensive animals were collected at day 7, post hypertonic saline injection. Equivalent amount of retina protein extracts (20 μg) were analyzed by Western blotting using a selective anti-phosphoSTAT3 (Tyr 705), anti-STAT3, and anti-β-actin antibodies. The band intensities were measured using enhanced chemiluminescence reagent and Versadoc imaging system. Bar graph represents mean data ±SEM. OH Eyes; ocular hypertensive eyes; OH Eyes + SNC-121, ocular hypertensive eyes with SNC-121 treatment. *p < 0.05; ****p < 0.0001; *n* = 6–9. In this experiment “n” represent a biological replicate.

**FIGURE 5:**
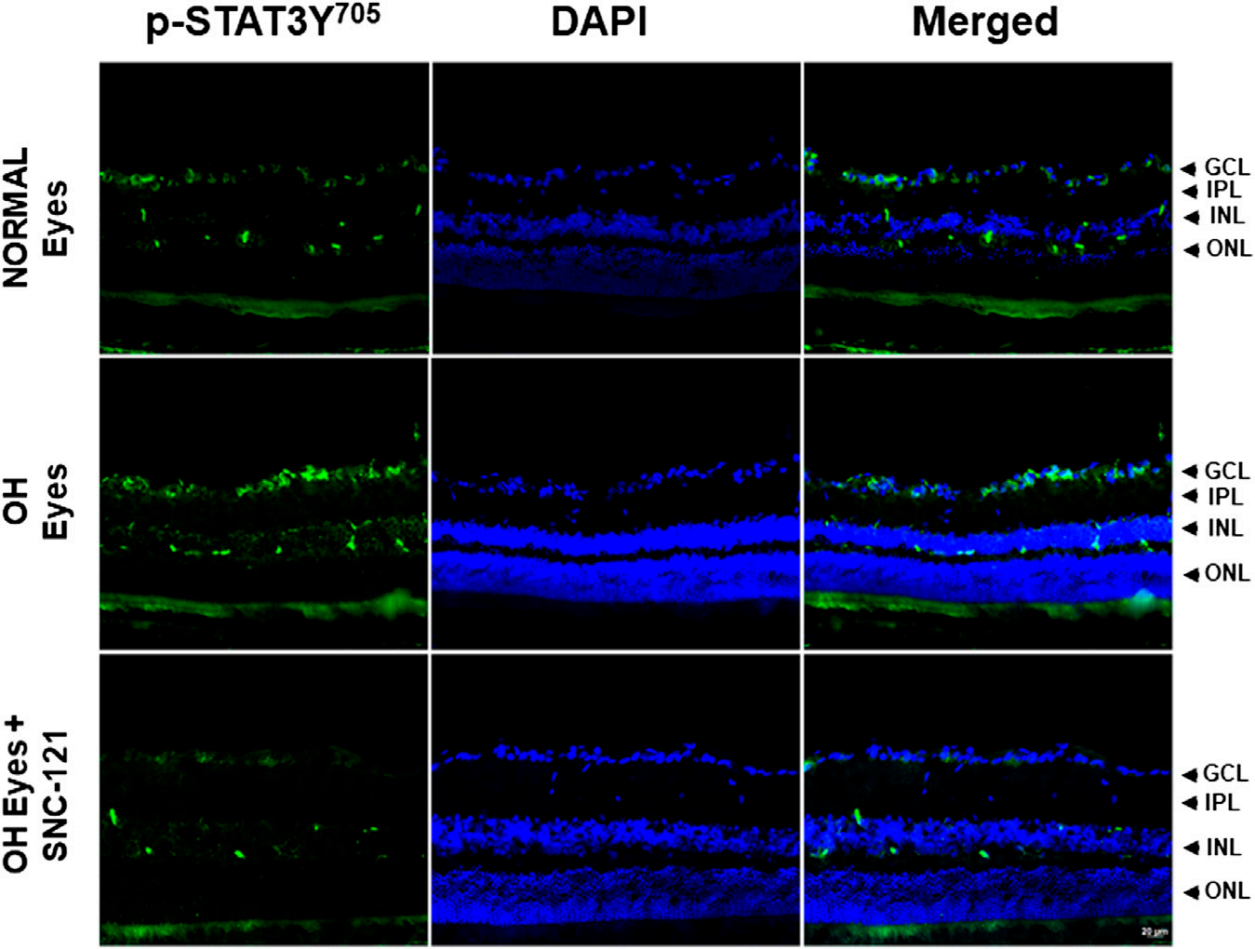
Changes in the signal transducer and activator of transcription 3 (STAT3) phosphorylation in response to ocular hypertension and δ-opioid receptor agonist (SNC-121) treatment. The retina from normal, ocular hypertensive, and SNC-121 treated ocular hypertensive animals were collected at day 7, post hypertonic saline injection. Retina cryosections were immunostained using a selective anti-phosphoSTAT3 (Tyr 705) antibody and the nuclei were counterstained with DAPI. Green color indicates staining for phosphorylation of STAT3 and blue for the nuclei. There was no positive immunostaining when primary antibodies were omitted (not shown). Data shown here is a representation of at least four independent experiments. We have used four animals in each treatment group. OH Eyes; ocular hypertensive eyes; OH Eyes + SNC-121, ocular hypertensive eyes with SNC-121 treatment, bar size 20 µm.

In non-ocular systems, studies have shown that phosphorylation of STAT3 at tyrosine 705 (Y705) activates and increases its transcriptional activity. To learn more about STAT3 and its regulatory roles in cytokines production, we measured phosphorylation of STAT3 at tyrosine 705. Our data provides evidence that phosphorylation of STAT3 at tyrosine 705 is significantly (*p* < 0.0001) increased in the retina of ocular hypertensive animals (Figure 4). Furthermore, we have seen 144 ± 17% (*p* = 0.02) increase in the total STAT3 protein expression in ocular hypertensive retina when compared with naïve retina (Figure 4). Data illustrated that SNC-121 treatment completely abrogated ocular hypertension-induced increase in STAT3^Y705^ phosphorylation. However, we were not successful to measure any appreciable phosphorylation of STAT1 and STAT2. We encountered technical issues with antibodies picking up numerous non-selective bands while used two antibodies from different sources. Based on our findings, we cannot fully ruled out the involvement of STAT1 and STAT2 in this process. Additional experiments are needed to determine the changes in total and phosphorylated STAT1 and STAT2 in normal and glaucomatous retina, which will be the goal of our future studies. To further identify the retina layers and cell-type in which STAT3 phosphorylation is predominantly increased, we performed immunohistochemistry using retina sections and a selective anti-phospho-STAT3^Y705^ antibodies in the normal, ocular hypertensive, and SNC-121 treated ocular hypertensive animals. As shown in Figure 5, phospho-STAT3 immunostaining was remarkably increased in retinal ganglion cell (GCL) and nerve fiber layers, however, a diffuse and punctate immunostaining was also seen in inner nuclear and outer plexiform layers of ocular hypertensive animals. The increase in phosphorylation of STAT3 at tyrosine 705 was drastically inhibited in SNC-121 treated animals specifically in GCL and nerve fiber layers.

### Effects of SNC-121 Treatment on STAT3 Acetylation

**FIGURE 6:**
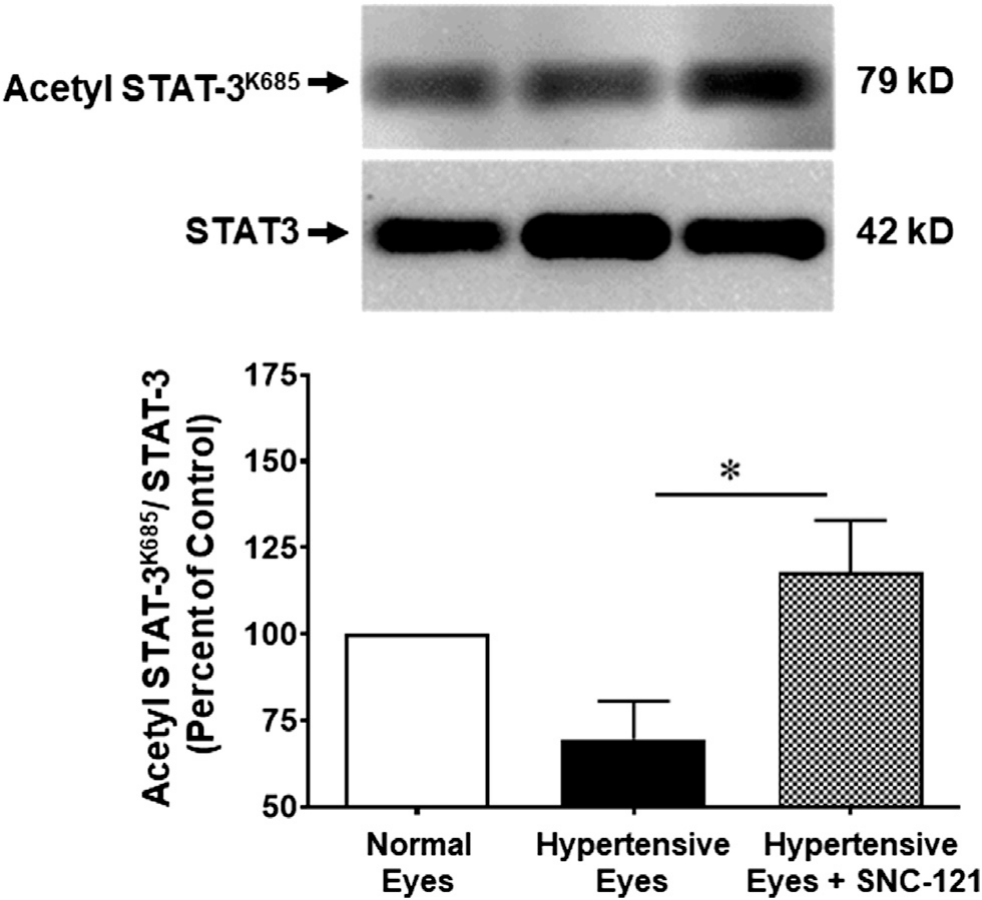
Changes in STAT3 acetylation at lysine 685 (AcK685) in response to ocular hypertension and SNC-121 treatment. The retina from normal, ocular hypertensive, and SNC-121 treated ocular hypertensive animals were collected at day 7, post hypertonic saline injection. Equivalent amount of retina protein extracts (20 μg) were analyzed by Western blotting using a selective anti-Acetyl STAT3 (AcK685) or anti-β-actin antibodies. The band intensities were measured using enhanced chemiluminescence reagent and Versadoc imaging system. Bar graph represents mean data ±SEM. OH Eyes; ocular hypertensive eyes; OH Eyes + SNC-121, ocular hypertensive eyes with SNC-121 treatment. *p < 0.05. *n* = 6, In this experiment “n” represent a biological replicate.

To establish a link whether transcriptional activity of STAT3 is regulated by phosphorylation, acetylation, or both, we have measured the acetylation of STAT3 using a selective acetyl STAT3 K685 antibody. This antibody recognizes only acetylated STAT3 at lysine 685 (AcK685). As shown in Figure 6, the acetylation of STAT3 was reduced as a result of glaucomatous injury. Moreover, we have seen a significant increase in the STAT3 acetylation at lysine 685 (AcK685) by SNC-121 treatment in ocular hypertensive animals. These data provide initial clues that protein acetylation of STAT3 will negatively regulate the transcriptional activity of STAT3, which may have reduced the activities of downstream signaling pathways including the production of pro-inflammatory cytokines.

### Inhibition of Pro-inflammatory Cytokines by a STAT3 Inhibitor in Rat Glaucoma Model

**FIGURE 7:**
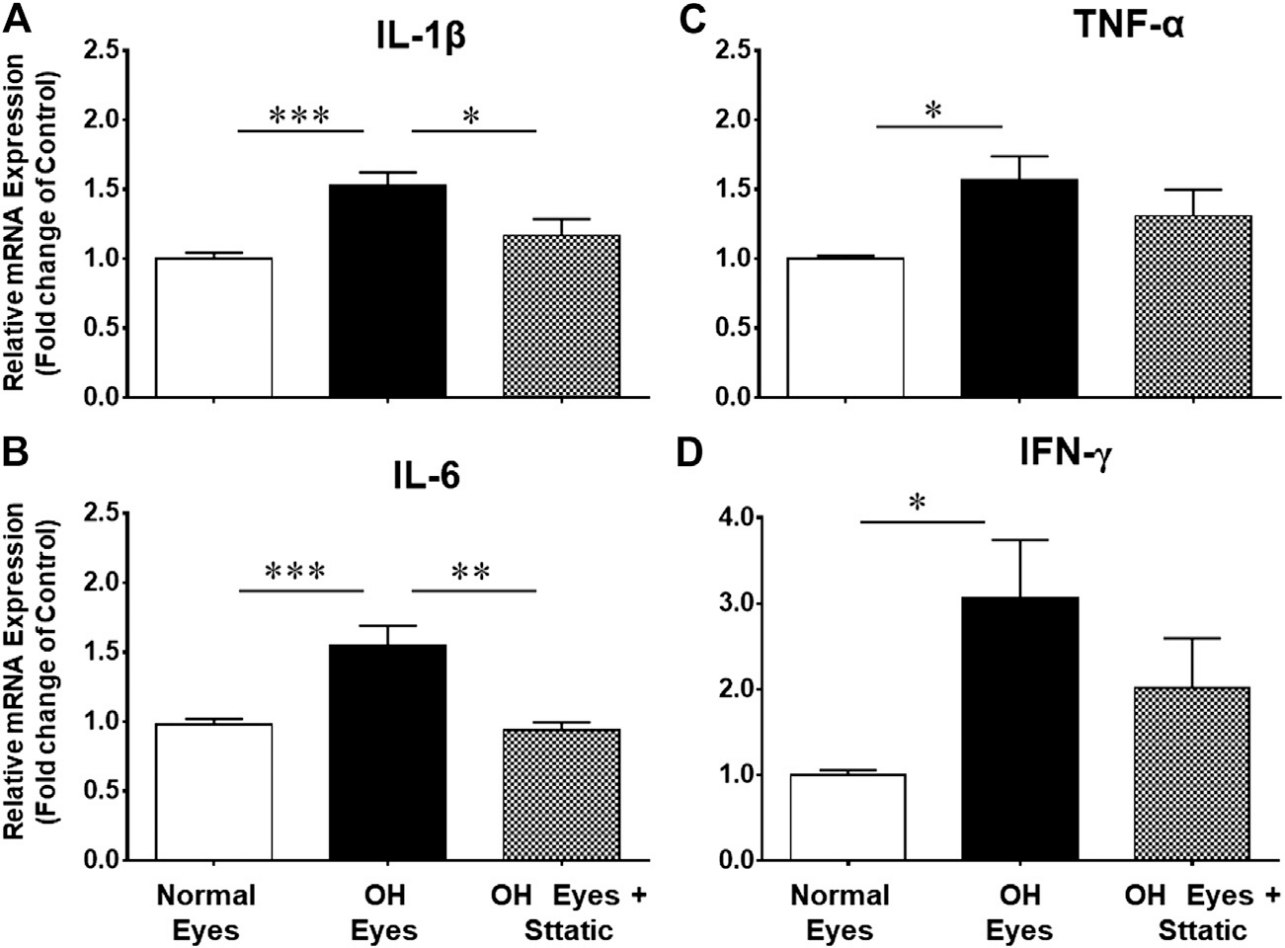
Inhibitory effects of Stattic, a selective STAT3 inhibitor, on the mRNA expression of IL-1β **(A)**, TNF-α **(B)**, IL-6 **(C)**, and IFN-γ **(D)**. The retina from normal, ocular hypertensive, and Stattic treated ocular hypertensive animals were collected at day 7, post hypertonic saline injection. Complementary DNA was synthesized from 1 µg total RNA extracted from retina. The relative changes in mRNA levels were measured by quantitative RT-PCR (qRT-PCR) using primers specific for each gene as indicated in Table 1. The qRT-PCR data was normalized using *ß*-actin gene expression as an internal control. The relative expression was calculated based on the comparative threshold cycle (2^−ΔΔCt^) method. Bar graph represents mean data ±SEM. OH Eyes; ocular hypertensive eyes; OH Eyes + Stattic, ocular hypertensive eyes with Stattic treatment. *p < 0.05; **p < 0.01; ***p < 0.001; *n* = 6–9. In this experiment “n” represent a biological replicate.

Stattic, a STAT3 inhibitor, was used to block the mRNA expression of pro-inflammatory cytokines. As shown in Figures 7A,B, Stattic administration (5 mg/kg, intraperitoneal, once a day for 7 days) fully attenuated the production of IL-1β and IL-6 in ocular hypertensive animals.

## Discussion

The current therapeutic modalities focus on lowering the elevated intraocular pressure (IOP), which is considered to be a primary risk factor for glaucoma (Boland and Quigley, 2007; Almasieh et al., 2012). However, patients undergoing glaucoma therapy continue to lose vision as a result of retinal ganglion cell (RGC) death suggesting IOP is not the only causative factor for RGC death in glaucoma. Primarily, RGCs die during glaucomatous injury but what causes the initiation of RGC degeneration is unknown. Numerous factors including neuroinflammation, epigenetic changes, biomechanical stress, neurotrophic factor deprivation, oxidative stress, and activation of glial cells (astrocytes and/or microglia) have been shown to be potentially involved in RGC death in glaucoma.

The eye is an “immune privileged” organ because peripheral immune cells are not able to cross blood-retinal barriers. Instead, the glial cells (e.g., microglia and astrocytes) are constituents of a dedicated neuroimmune system and provide immune surveillance. In physiological conditions, the glial cells provide both pro- and anti-inflammatory cytokines and they play crucial roles in normal and disease condition including phagocytosis, free radical reduction, cellular repairs, and maintaining metabolites to provide energy (Grieshaber et al., 2007; da Fonseca et al., 2014; Sanderson et al., 2014). However, under pathological conditions the excessive release of pro-inflammatory cytokines and reactive oxygen species may cause synaptic dysfunction, disruption of axonal transport, and ultimately RGC death. Reactive astrocytes can secret a variety of cytokines and chemokines including CCL2, CXCL1, CXCL10, GM-CSF, and IL-6 that may further activate microglia, infiltrating dendritic cells, monocytes/macrophages and T cells in the inflamed tissues (Liddelow et al., 2017). Studies have also shown that mechanical strain due elevated IOP leads to the activation of NLRP3 inflammasome components *IL-1*β*, NLRP3, ASC*, and *CASP1* in rat and mouse retinas (Albalawi et al., 2017). As a result, an imbalance in pro- and anti-inflammatory cytokines will develop neuroinflammation that will slowly and progressively damage RGCs. In neurodegenerative diseases, neuroinflammation has been shown to be regulated by glial cells (astrogliosis and microglial activation), elements of blood brain barriers, and systemic inflammatory process (immune cells) that may be harmful or beneficial depending upon the severity and the duration of the neuroinflammation. As a result, neuroinflammation has been shown to play crucial roles in numerous neurodegenerative diseases including Alzheimer’s disease, Atrophic Lateral Sclerosis, and glaucoma (Husain et al., 2012a; Calsolaro and Edison, 2016; Ransohoff, 2016; Liu and Wang, 2017; Williams et al., 2017).

In this manuscript, we have shown a significant increase in mRNA expression of pro-inflammatory cytokines and their associated signaling molecules including IL-1β, TNF-α, Fas, IL-6, LIF, and IFN-γ in response to glaucomatous injury, which was blocked by SNC-121 treatment. We chose 1 mg/kg body weight of SNC-121 based on our previous studies using a broad range opioid agonist and found 1 mg/kg body weight provide optimal neuroprotection (Husain et al., 2009). Additionally, we evaluated multiple doses of SNC-121 (0.01–10 mg/kg body weight) for RGC neuroprotection and found that 1–10 mg/kg doses of SNC-121 provide similar amount of RGC neuroprotection. Considering the addictive nature of opioids, we chose lower dose (1 mg/kg body weight) in all of our previous and current studies. Additionally, we did not find any additional beneficial effects if animals were treated with SNC-121 for up to 28 days (once a day) so we kept our drug treatment regimen to 7 days (Abdul et al., 2013). As a result of systemic treatment of SNC-121 for 7 days, we did not see any changes in the blood pressure, IOP, and body weight when compared with vehicle treated animal. Furthermore, we have shown that elevated protein expression of TNF-α (Abdul et al., 2013), IL-1β, and IL-6 were attenuated by SNC-121 treatment in ocular hypertensive animals. These data provide initial clues that elevated levels of pro-inflammatory cytokines that have been seen in glaucomatous retina is an early event. It important to emphasize that we have seen reduction of mRNA expression of Fas and LIF while their protein expressions were not measured in this study. We do understand that changes in mRNA expression may not always correspond to the changes in protein levels. Further studies will be required to confirm if SNC-121 treatment also affect the protein levels of Fas and LIF. In chronic glaucoma rat model, we have shown the loss of RGCs and their function (measured by Pattern Electroretinograms) at 14 days, post glaucomatous injury (Abdul et al., 2013), which indicates that production of pro-inflammatory cytokines preceded any RGC loss during glaucoma progression. Additionally, we have shown that SNC-121 treatment for 7 days reduces the production of pro-inflammatory cytokine (TNF-α), improved RGC function (as measured by Pattern ERGs), and reduced the loss of RGC numbers (as measured by retrograde labeling of RGCs) in rat glaucoma model (Abdul et al., 2013). Earlier, we have demonstrated that SNC-121 actions were fully blocked by Naltrindole, a selective δ-opioid receptor antagonist (Akhter et al., 2013b; Husain et al., 2014; Zaidi.et al., 2020).

Similar to our data, studies have also shown that dysregulation of cytokine signaling in the RGC projections in DBA/2J mice was seen before IOP induction and axonal loss (Wilson et al., 2015). These findings prompted us to believe that activities of certain transcription factors may have been increased during the early stages of glaucoma. To shed more light on this idea, we measured the changes in the mRNA expression of transcription factors STAT1, STAT2, and STAT3 and found that mRNA levels of all STATs were increased in the retina of ocular hypertensive animals. Moreover, phosphorylation of STAT3 at tyrosine 705 was increased, which is known to be required for its activation and transcriptional activity. Intriguingly, STAT3 mRNA expression and phosphorylation were fully blocked by SNC-121 treatment. Based on these data, we believed that phosphorylation of STAT3 at tyrosine 705 is critical and plays a crucial role in the production of certain pro-inflammatory cytokines. This idea was further supported by the data that mRNA expression of IL-1β and IL-6 were completely inhibited by a selective STAT3 inhibitor, Stattic. Contrary to this, TNF-α and IFN-γ were partially inhibited by Stattic, suggesting that production of interleukins are mediated through STAT3 dependent pathways whereas other transcriptional factors such as p53, NF-ĸB, and c-fos could be involved in the production of other cytokines. Increases in IL-6 and JAK/STAT have been shown in early pressure-induced optic nerve head injury (Johnson et al., 2011). Conversely, IL-6 released from glial cells has been shown to protect RGCs in response to hydrostatic pressure induced apoptotic RGC death (Sappington et al., 2006). Earlier, we have shown that activation of δ-opioids receptors suppressed “redox-sensitive” transcriptional factor, nuclear factor-kappa B (NF-ĸB) in optic nerve head astrocytes (Akhter et al., 2013a). Proteomic analysis of glaucomatous retina obtained from human and experimental animal models also showed an up-regulation of receptor-interacting protein kinase (RIPK) that are also involved in the regulation of NF-ĸB (Tezel, 2013). Furthermore, studies have shown that antioxidant treatment can reduce NF-ĸB and neuroinflammation and provide neuroprotection in glaucoma model (Yang et al., 2016). Taken together, previous studies have shown that NF-ĸB plays key roles in glaucoma pathology (Kitaoka et al., 2006; Akhter et al., 2013a). However, we have not evaluated its direct involvement in the regulation of pro-inflammatory cytokines in the current study.

As a result of neuroinflammation, numerous downstream signaling pathways (e.g., cytokines-dependent proteins, caspases, and pro-apoptotic proteins) could also be up-regulated and facilitate the RGC death in glaucoma. We have shown a significant increase in Fas expression in ocular hypertensive animals. Fas is a constitutively expressed 45 kDa membrane bound protein that belongs to the TNF-α receptor superfamily (Watanabe-Fukunaga et al., 1992). FasL is a ligand for Fas, which is expressed in numerous activated T cells (Suda et al., 1995) and glial cells (Lee et al., 2000; Krishnan et al., 2019). The Fas/FasL system is essential for homeostasis of the immune system and its impairment can lead to neuroinflammation. Studies have also shown that pro-inflammatory cytokines can also increase the expression of Fas (Kondo et al., 2009). Our data shows an increase in Fas expression, which could be as a result of increased production of pro-inflammatory cytokines. Mechanistically, pro-inflammatory cytokines may facilitate Fas-mediated apoptosis of RGCs. Increased levels of both pro-inflammatory cytokines and Fas in the retina of ocular hypertensive animals indicated that these pro-inflammatory cytokines may have facilitated RGC death by caspases activation and apoptosis in Fas-dependent pathways as shown earlier in other systems (Ye et al., 2019). Our data showed that proinflammatory cytokines are inhibited by SNC-121 treatment, suggesting that δ-opioid agonist are potential agents to regulate neuroinflammation and RGC death in glaucoma. Earlier, we have shown that glaucomatous injury increased the protein expression of TNF-α, caspases-3, and 8 and all of them were blocked by a broad-range opioid agonist, Morphine (Husain et al., 2012a).

Data illustrated an increase in the leukemia inhibitory factor (LIF) and IL-6 in ocular hypertensive animals. LIF is a multi-functional cytokine which belongs to the IL-6 superfamily. IL-6 superfamily comprised of oncostatin M (OSM), IL-6, IL-11, ciliary neurotrophic factor (CNTF), and cardiotrophin-1 (CT-1) (Taupin et al., 1998; Turnley and Bartlett, 2000). LIF binds to its specific receptors LIFR, then recruit gp130 to form a high affinity receptor complex to induce the activation of the downstream signaling pathways such as JAK/STAT3, PI3K/Akt, ERK, and mTOR pathways (Burdon et al., 2002; Pera and Tam, 2010). We have shown that both LIF and STAT3 mRNA expression and STAT3 phosphorylation at tyrosine 705 were increased in the ocular hypertensive animals, which were fully blocked by SNC-121 treatment, suggesting that LIF might be required for the STAT3 activation for pro-inflammatory cytokines production. However, we did not measured the association of LIF with gp130 and JAK/STAT3 in the current study and this will be the goal for our future studies.

Recently, we have also shown that a sustained activation of δ-opioid receptors can lead to epigenetic changes via regulating histone acetylation and histone deacetylates (HDACs) in optic nerve head astrocytes and rat glaucoma model (Zaidi et al., 2020) and *unpublished results*). Considering our latest findings, we measured if δ-opioids-mediated epigenetic changes can regulate neuroinflammation in glaucoma. We hypothesized that protein acetylation provides RGC neuroprotection. We provide a mechanistic link that acetylation of STAT3 is reduced in response to glaucomatous injury when it was normalized with total STAT3. Conversely, we have seen a significant increase in the acetylation of STAT3 at lysine 685 (Ac-K685) by SNC-121 treatment in ocular hypertensive animals. Mechanistically, we proposed that δ-opioids will reduce HDACs and NF-ĸB activities and simultaneously increased acetylation of STAT3. Studies have shown that transcriptional activities of numerous transcription factors including STAT3 are regulated by lysine acetylation, which is regulated by HDACs. More recently, studies have shown that transcriptional factor NF-ĸB-mediated cytokines production is inhibited by HDAC inhibitors via acetylation-mediated NF-ĸB transcriptional activity (Kramer et al., 2006; Leus et al., 2016). Recently, we also have shown that reduced acetylation of Foxo1 (transcription factor) in hybrid Th1/17 cells results in its enhanced transcriptional activity (Chatterjee et al., 2018). These unique epigenetic-mediated signaling mechanisms play pivotal roles in non-ocular systems, however, if such transcription dysregulation exist in glaucoma remains largely unknown. We speculate existence of similar molecular mechanisms for δ-opioid-induced acetylation of STAT3 in the retina. In other systems, studies have shown that inhibition of HDACs enhances STAT acetylation, blocks NF-ĸB, and suppressed the production of pro-inflammatory cytokines (Kumar et al., 2017). In addition, HDAC inhibitor (sodium butyrate) attenuated STAT1-HDAC1/2 complex formation and simultaneously increased STAT1 acetylation. Increased acetylation of STAT1 increased its physical interaction with transcription factors (NF-ĸB or others), thereby inhibiting downstream signaling and production of pro-inflammatory cytokines (Feng et al., 2016; Li et al., 2016; Kumar et al., 2017). Data presented in the current manuscript support our hypothesis that protein acetylation regulates the activity of transcription factor STAT3, which will subsequently negatively regulate the production of pro-inflammatory cytokines (IL-1β and IL-6). Data also provide concrete evidence that δ-opioids regulate neuroinflammation via STAT3 dependent pathway in which protein acetylation played a regulatory role in the production of selective pro-inflammatory cytokines (e.g., IL-1β and IL-6), however, other transcription factors such as NF-ĸB may also be involved directly or indirectly in the regulation of cytokines. We cannot ruled out the anti-inflammatory roles of other non-opioid agents during glaucoma pathology.

Taken together, data presented in this manuscript provide evidence that numerous pro-inflammatory cytokines and their associated signaling molecules are up-regulated in response to glaucomatous injury. These cytokines are up-regulated at early stages of glaucoma and even prior to any RGC loss. Additionally, transcription factor (STAT3) activation and phosphorylation at tyrosine 705 is significantly increased in ocular hypertensive animals. In contrast, STAT3 acetylation at lysine 686 (Ac-K685) was not reduced by glaucomatous injury but significantly increased by δ-opioid receptor activation. Moreover, all pro-inflammatory cytokines and STAT3 phosphorylation were fully blocked by SNC-121. Intriguingly, a selective inhibitor of STAT3, Stattic, fully blocked the production of IL-1β and IL-6 but it had a partial inhibitory effect on TNF-α and IFN-γ, suggesting the involvement of other transcriptional factors.

## Conclusion

In conclusion, data presented in the current manuscript support the idea that multiple neuroinflammatory genes and pathways are involved in the development of glaucoma. However, more work is needed to determine when and how these genes and pathways are activated. It is also equally important to know which genes and pathways are neuroprotective or pathogenic in glaucoma. The current study provides novel insights that pro-inflammatory cytokines such as IL-1β and IL-6 are regulated by δ-opioids via STAT3-dependent pathways in rat glaucoma model. The understanding of the regulatory mechanisms involved in components of inflammatory responses will be highly desired to develop a better therapy that can target neuroinflammation. This information would then guide future therapeutic strategies, which would include the use of selective inhibitors to target pro-inflammatory cytokines or its upstream regulators to treat glaucoma patients.

Opioid receptor agonists (e.g., morphine and buprenorphine) are widely used in clinics for pain management. However, chronic use of opioids can also results in unwarranted side effects (e.g., addiction and withdrawal). However, δ-opioids have lesser side effects as compared to broad range non-selective opioids. Alternatively, side-effects of opioids can be overcome by local application (e.g., topical or intravitreal) vs. systemic application. Nevertheless, the therapeutic potential of δ-opioid agonists have been shown in preclinical models for other conditions including Parkinson’s disease, pain management, and depression. Based on our data, we strongly believe that δ-opioid agonists represent to have a high potential for glaucoma therapy.

## Data Availability

The raw data supporting the conclusions of this article will be made available by the authors, without undue reservation.
